# Targeting GPR3 as a novel approach for nicotine cessation therapeutic development

**DOI:** 10.1038/s41386-025-02202-3

**Published:** 2025-08-27

**Authors:** Allison S. Mogul, Kendyl N. Laumann, Malia Bautista, JP Fowler, Bruce E. Blough, Elaine A. Gay, Christie D. Fowler

**Affiliations:** 1 Department of Neurobiology and Behavior, University of California Irvine, Irvine, CA USA; 2 Center for Drug Discovery, RTI International, Research Triangle Park, Durham, NC USA

**Keywords:** Addiction, Pharmacology, Target validation

## Abstract

Tobacco use remains the leading cause of preventable death worldwide. Unfortunately, currently available cessation aids have limited long-term efficacy. GPR3 is a Gαs coupled receptor expressed in discrete brain regions, with notably high expression in cholinergic neurons of the medial habenula. Here, we investigated whether modulation of GPR3 could be a viable target for therapeutic development to promote nicotine cessation. We first examined whether our recently developed GPR3 receptor agonist, RTI-19318-32, could induce effects on intravenous nicotine self-administration at low, moderate or high nicotine doses in mice. We found that in both males and females, RTI-19318-32 significantly reduced nicotine intake at all self-administered nicotine doses, thereby supporting the validity of this therapeutic approach for individuals using varying levels of daily nicotine. RTI-19318-32 was further validated as being selective for GPR3, as it did not alter nicotine intake in GPR3 knockout mice, nor did it exert effects on anxiety-associated behavior or locomotion. While the higher RTI-19318-32 dose attenuated food-related reinforcement behavior, it was ineffective in altering baseline food consumption. Moreover, the lower RTI-19318-32 dose did not alter food reinforcement behavior, indicating selectivity in mediating nicotine intake. Finally, GPR3 expression co-localized with multiple nAChR subunits in the medial habenula, thereby supporting our proposed targeted approach for circuit engagement intentionally directed at modulating the drive to consume nicotine. Taken together, these data reveal the functional significance of agonist-inducted activation of the GPR3 receptor and establish the validity of focusing on therapeutic development of GPR3 ligands for nicotine cessation.

## Introduction

Tobacco and nicotine products continue to be the leading cause of preventable disease worldwide, leading to an estimated 7 million deaths per year [1, 2]. Approximately 68% of smokers report wanting to quit, however, few are successful in the long term [3]. The vast majority of nicotine products infer a high addiction liability, including tobacco cigarettes, electronic cigarettes, quick-dissolving nicotine lozenges, and nicotine pouches, among others. Electronic cigarette use in particular has increased in popularity over the last decade, expanding the use of nicotine products to many never-smokers of tobacco products [4, 5], and electronic cigarette use has been associated with an increased risk of future initiation of tobacco smoking [6]. A recent report indicates that a majority of American adults who use electronic cigarettes want to quit [7]. First line therapeutics for nicotine product cessation include nicotine replacement therapy (NRT), varenicline, and bupropion [8]. These therapeutics, either used alone or in combination, can be helpful for those struggling to quit, however, in clinical trials, the most successful cessation rates are only ~20% or less after 1 year [9]. Given that nicotine has been proposed to induce changes in brain function to lead to dependence via actions on the nicotinic acetylcholine receptors (nAChRs), it is possible that continuing to pharmacologically activate the nAChRs (e.g., with NRT or varenicline) may perpetuate and/or sustain the changes in neurocircuit function that underlie the addictive state, which thereby highlights the need for alternative directed approaches.

G-protein coupled receptors (GPCRs) can be effective drug targets due to their expression patterns, location on the cell membrane and diverse second messenger signaling profiles [10]. G-protein coupled receptor subtype 3 (GPR3) is an orphan GPCR with evidence of constitutive Gαs signaling [11]. Lipid structures, specifically oleic acid, have recently been shown to interact with the GPR3 receptor in vitro [12], but it remains unknown if oleic acid mediates receptor function at endogenous levels in the brain in vivo. In adulthood, GPR3 is expressed in various regions of the central nervous system (CNS) that have been implicated in drug reinforcement and reward, including the medial habenula [13].

Given the function of the medial habenula in mediating nicotine reinforcement [13–16], our recent development of GPR3-specific agonists [17] and identified expression of GPR3 in cholinergic habenular neurons, we sought to examine whether modulation of GPR3 could mediate nicotine reinforcement and intake. First, we assessed the effects of the GPR3 agonist on intravenous self-administration across a range of low, moderate and high nicotine doses. Next, male and female mice lacking the GPR3 receptor or wildtype controls (GPR3^-/-^ or GPR3^+/+^, respectively) were examined with intravenous nicotine self-administration to further probe the role of the GPR3 receptor on nicotine intake. To further support these findings, we replicated the effects of the GPR3 agonist on self-administration in wildtype GPR3^+/+^ mice and further validated the GPR3 agonist in GPR3^-/-^ mice. Finally, cell-type specific expression of GPR3 in the medial habenula was examined with single cell sequencing data to determine co-expression patterns. Taken together, these findings identify GPR3 as a novel, viable target for nicotine cessation therapeutic development.

## Methods

### Animals

GPR3^-/-^ mice were generously donated by Dr. George Kunos at the National Institute on Alcohol Abuse and Alcoholism at the National Institutes of Health [18], and experimental GPR3^-/-^ mice and wildtype GPR3^+/+^ littermates were derived from breeding heterozygous GPR3^+/-^ male and female mice on a C57BL/6J background. Genotyping was performed from tail clippings using the reverse primer (GGAATTAAGCCCTGGTGGACCTAAC) and forward primers (TATCCACTCTCCAAGAACCATCTGG and GGGCCAGCTCATTCCTCCCACTCAT). C57BL/6J mice were purchased from Jackson Labs or bred within the laboratory colony. All mice were maintained in an environmentally controlled vivarium on a 12 h reversed light/dark cycle, and experiments began at ~7 weeks of age. Experiments were conducted in accordance with the University of California Irvine Institutional Animal Care and Use Committee.

### Drugs

The GPR3 agonist, RTI-19318-32, was synthesized as previously described (compound 32) [17]. We previously established that the 3-trifluoromethoxy analog was found to be the most potent, full agonist with an EC_50_ of 260 nM and 90% efficacy [17]. RTI-19318-32 (1 or 10 mg/kg) was prepared fresh daily in vehicle (10% DMSO, 10% Tween-80, 80% saline (0.9% sodium chloride) solution), and the pH was adjusted to 7.4. The drug was injected intraperitoneally at a volume of 1 ml/kg. (−)-Nicotine hydrogen tartrate salt was purchased from MP Biomedicals, dissolved in saline solution, and adjusted to pH 7.4. All doses of nicotine refer to the free-base form.

### Nicotine intravenous self-administration

Mice were mildly food-restricted to 85–90% of their free-feeding weight and were then trained to lever press for food pellets (grain-based, 20 mg, 5TUM, TestDiet) with a two-lever operant task across ascending fixed ratio (FR) schedules from one up to five lever presses, as previously described [19]. At the start of the session, both levers were extended into the chamber and remained present throughout the daily 1 h session. Responses on the active lever that met the FR criteria resulted in the delivery of a food pellet, which was paired with a small LED cue light for a 20 s timeout period. The resulting final reinforcement schedule of FR5TO20 was imposed across food training sessions 4–7. Responses on the inactive lever were recorded but had no scheduled consequences. Testing was conducted 6–7 days per week, and behavioral responses were recorded with a MedPC interface (Med Associates). After food training, subjects were anesthetized with isoflurane and catheterized as previously described [14, 20]. The catheter tubing was passed subcutaneously from the animal’s back to the right jugular vein, and a 1 cm length of catheter tip was inserted into the vein and tied with surgical silk suture. Following surgery, animals were given ≥72 h to recover, and were then provided 1 h access to reestablish food responding under the FR5TO20 sec schedule, until the criteria of >25 pellets/session was achieved for three consecutive sessions. Mice were then transitioned to respond for intravenous nicotine self-administration under the FR5TO20 sec schedule of reinforcement during 1 h daily sessions, 6–7 days per week, at the training dose of nicotine (0.03 mg/kg per infusion). The solution was delivered through tubing into the intravenous catheter by a Razel syringe pump (Med Associates). Based on prior findings [14, 19], mice typically achieve stable responding for nicotine after ∼5 days of acquisition, which can be evidenced by <20% variability in responding between consecutive sessions. All mice were provided access to the acquisition dose of nicotine for 8 days to allow for consistency in the total number of sessions. After achieving stable responding on the 0.03 mg/kg per infusion dose, mice were transitioned to the moderate dose of 0.1 mg/kg per infusion nicotine for 5 days. This dose results in a similar level of drug intake as that found at higher doses given behavioral titration via self-administration [19]. To obtain the full dose response function, the mice were provided access to either 0.03, 0.1, 0.25 or 0.4 mg/kg/infusion dose for 5 days, and then reestablished at baseline on 0.1 mg/kg per infusion for at least 2 days in between each dose, this pattern was repeated until mice had access to all of the doses. The mean of the final 3 days on each dose was calculated for each subject. Finally, for dosing of the GPR3 agonist, RTI-19318-32, mice were administered 0, 1, or 10 mg/kg intraperitoneally 20 min prior to the session, counterbalanced in a Latin square manner across sessions with at least 2 days of baseline responding in between each dose. Catheters were flushed daily with physiological sterile saline solution (0.9% w/v) containing heparin (100 units/ml). Catheter integrity was verified with the ultra-short-acting barbiturate anesthetic Brevital (2%, methohexital sodium, Eli Lilly) at the end of the study.

### Food self-administration and feeding studies

For operant food self-administration, mice were food restricted as described above. After stable responding was achieved across 3 consecutive sessions, mice were administered the GPR3 agonist, RTI-19318-32, at doses of 0, 1, or 10 mg/kg intraperitoneally 20 min prior to the session, counterbalanced in a Latin square manner across sessions and with at least 2 days of baseline responding in between each dose. Behavioral responses were recorded with a MedPC interface (Med Associates). For the GPR3 knockout mice, subjects were food trained as above, and to obtain reinstated feeding following food restriction, mice were provided full food in the home cage and weighed daily. To assess the effects of the GPR3 agonist, RTI-19318-32, on food consumption with free feeding conditions, wildtype male and female mice were habituated to daily sessions of 2 h of free-feeding in single housed cages, and the amount of food consumed and body weight were recorded after each session by an experimenter blinded to the injection condition. With this feeding pattern, mice maintained daily body weights above 90% of their baseline weight. On the 5th day of this schedule, mice were pretreated with either vehicle or 10 mg/kg RTI-19318-32 in a counterbalanced design, 20 min prior to the feeding session. On the 7th day, subjects were administered the counterbalanced dose of either vehicle or 10 mg/kg with food consumed and body weight recorded after the 2 h free feeding session.

### Open field locomotor test

The open-field chamber was composed of plexiglass (35 cm L × 35 cm W × 31 cm H). The GPR3 agonist, RTI-19318-32, was administered intraperitoneally 20 min prior to subjects being placed in the chamber. Subjects were scored in the open-field apparatus for a 15-min test to assess locomotor activity, which began 5 min after being placed in the arena to allow for a habituation period. Activity was recorded with a video camera and scored by two experimenters blinded to the group condition with ANY-Maze Software (Stoelting Co., Wood Dale, IL, USA).

### Elevated plus maze

The elevated plus maze (EPM) was composed of 4 opaque runways 5 cm wide and 35 cm in length, which were elevated 40 cm from the floor. Two opposing closed runways had opaque walls 15 cm in height, whereas the other two opposing sides did not contain walls (open arms). Subjects were injected with the GPR3 agonist, RTI-19318-32, 20 min prior to being placed in the center portion of the elevated plus maze, and behavior was recorded for 5 min with a video camera. Behavior was scored by two experimenters blinded to the group condition with ANY-maze software.

### MERSCOPE

**Fig. 1:**
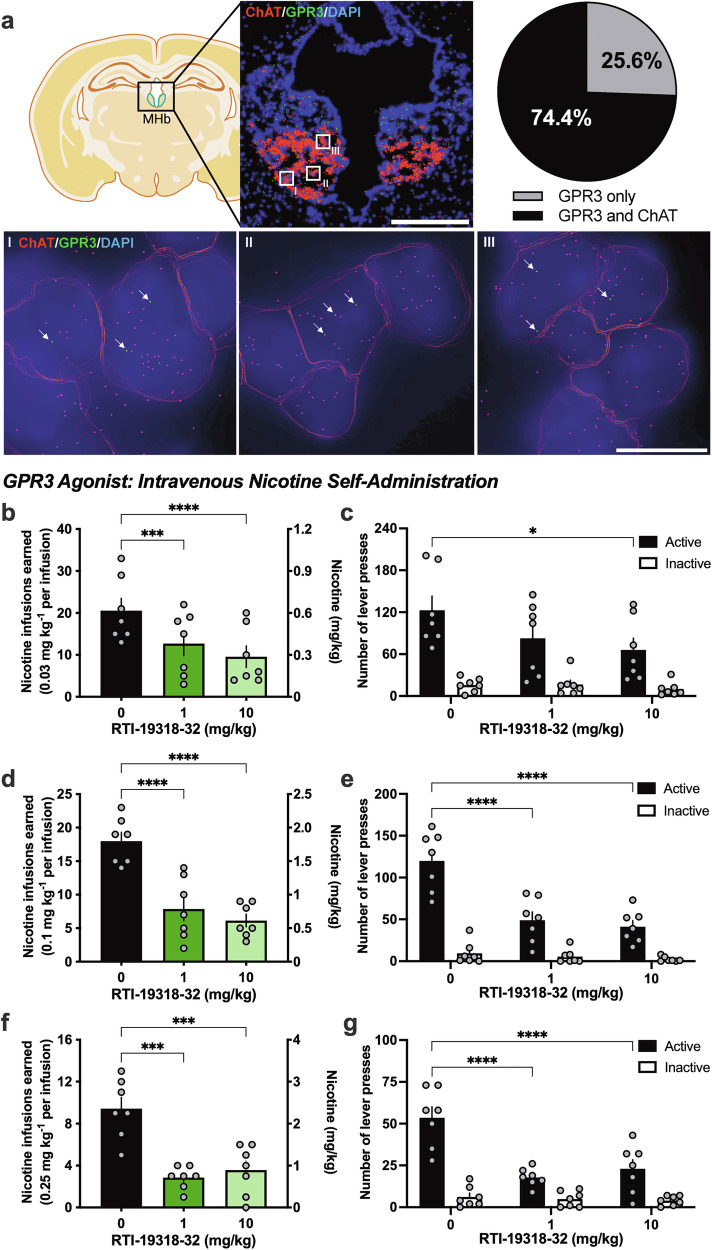
Administration of a GPR3 agonist attenuates nicotine intake. **a** GPR3 is expressed in cholinergic neurons of the ventral medial habenula. Top left panel: Representative image of coronal mouse brain slice, with the medial habenula outlined in green. Top middle panel: MERSCOPE image of medial habenula displaying ChAT (red) and GPR3 (green) transcripts. Scale bar = 500 μm. Top right panel: Quantification of GPR3 cells co-expressing ChAT from Vizgen’s publicly available MERSCOPE datasets (n = 3 female mice, 6–8 weeks of age) reveals that the majority of the GPR3 positive cells co-localize with ChAT. Bottom panels I, II and III: Higher magnification images of the denoted areas I, II and III from the white square outlines in the top middle panel. Cells shown express GPR3 transcripts (green), ChAT transcripts (red) and DAPI (blue). White arrows denote the GPR3 mRNA puncta. Scale bar = 10 μm. **b**–**g** Reduced nicotine self-administration following administration of the GPR3 agonist, RTI-19318-32, in mice. **b** At the low dose of self-administered nicotine (0.03 mg/kg/infusion; n = 7 male mice), both the 1 and 10 mg/kg doses of RTI-19318-32 significantly decreased nicotine intake. Graph shows both the number of nicotine infusions earned (left y-axis) and total mg/kg nicotine consumed (right y-axis). **c** Active lever pressing was selectively reduced with administration of the 10 mg/kg dose of RTI-19318-32, and no differences were found with inactive lever pressing. **d** At the moderate dose of self-administered nicotine (0.1 mg/kg/infusion; n = 7 male mice), administration of the 1 and 10 mg/kg doses of RTI-19318-32 significantly decreased nicotine intake. The graph shows both the number of nicotine infusions earned (left y-axis) and total mg/kg nicotine consumed (right y-axis). **e** Active lever pressing was selectively reduced at both the 1 and 10 mg/kg RTI-19318-32 doses, with no differences for inactive lever pressing behavior. **f** At the high dose of self-administered nicotine (0.25 mg/kg/infusion; n = 7 male mice), significant reductions in the number of nicotine infusions earned were found for both the 1 and 10 mg/kg RTI-19318-32 doses. The number of nicotine infusions earned (left y-axis) and total mg/kg nicotine consumed (right y-axis) are shown. **g** RTI-19318-32 selectively reduced lever pressing behavior at the active, but not at the inactive lever. Data represented as mean ± SEM; all individual data points shown on bar figures. *p < 0.05, ***p < 0.001, ****p < 0.0001.

MERSCOPE images and co-localization quantification was conducted through the publicly available dataset, Vizgen MERFISH Mouse Receptor Map (https://info.vizgen.com/mouse-brain-map). Datasets accessed included three subjects containing the medial habenula (coronal section, slice 2) from female C57BL/6J mice at 6–8 weeks of age. The data consisted of a 438 gene panel, stained images and cell segmentation boundary coordinates. The data was visualized using Seurat and R Studio. GPR3 and choline acetyltransferase (ChAT) transcript co-localization was quantified by experimenters blinded to the gene conditions (Fig. 1).

### Single cell spatial transcriptomics

Single cell transcriptomic data was obtained from a separate open-source database published by Macosko and colleagues at the Broad Institute (accessible at: https://singlecell.broadinstitute.org) [21] (Fig. 5). The dataset containing the medial habenula included 19 mice (7 males, 12 females). Forty-four clusters containing a total of 33,416 cells were characterized as medial habenular neurons based on transcriptional markers. From this dataset, four groups were created based on expression of GPR3 and ChAT: Co-expressing cells (Gpr3 and ChAT, transcript number >0; denoted as GPR3^+^/ChAT^+^), Gpr3 only (Gpr3 > 0, ChAT = 0; denoted as GPR3^+^), ChAT only (Gpr3 = 0, ChAT > 0; denoted as ChAT^+^), or neither (Gpr3 = 0, Chat = 0). Expression of the nicotinic acetylcholine receptor subunits *Chrna3* (α3), *Chrna4* (α4), *Chrna5* (α5), *Chrna6* (α6), *Chrna7* (α7), *Chrnb2* (β2), *Chrnb3* (β3), or *Chrnb4* (β4) mRNA transcripts were then determined for each category. Percent expression was calculated by dividing the number of cells containing a given nAChR subunit transcript by the total cells in that category. No significant sex differences were found in GPR3-expressing cells (Fig S1 and S2), so data were combined from both males and females. It should be noted that a statistically significant sex difference was found in the in the α5-expressing ChAT^+^ (GPR3^-^) cells, which represented a minor population of this cellular phenotype (<10% of all ChAT^+^ (GPR3^-^) cells) (Fig S2). Additional control analyses of the medial habenula cells examined the co-expression of GPR3 with estrogen receptor: *Esr1* (estrogen receptor α), *Esr2* (estrogen receptor β), and Gper1 (G protein-coupled estrogen receptor) (Fig S1d).

### RT-qPCR

The medial habenula was dissected and then flash frozen and stored at −80 °C until further processing. RNA was extracted from homogenized tissue and the quality of the RNA was determined by a NanoDrop 2000 spectrophotometer (ThermoScientific) [20]. For each sample, 100 ng of total RNA was reverse transcribed into cDNA with the iScript cDNA synthesis kit (Bio-Rad Laboratories, catalog # 1708890). TaqMan Universal Master Mix II was used with real time PCR gene expression assays for *Gpr3* (Thermo Fisher, catalog #00433719) and *Actb* (Thermo Fisher, catalog # 4331182). Samples were tested in duplicate and quantified with a CFX96 RT-qPCR system (Bio-Rad). Normalized gene expression (2^ΔCt^) was multiplied by 100.

### Statistical analyses

Data were analyzed by a t-test, one-way or two-way ANOVA with Prism 10 software (GraphPad, La Jolla, CA, USA), as appropriate. Significant main or interaction effects were followed by Sidak post-hoc comparison with correction for multiple comparisons. The criterion for significance was set at α = 0.05.

## Results

### GPR3 agonist treatment decreases nicotine self-administration

We first examined whether GPR3 is co-localized in cholinergic neurons in the medial habenula with spatial transcriptomics (Fig. 1a). We found that the majority of GPR3 expressing cells (74.4% ± 1.6, mean ± SEM) also express choline acetyltransferase (ChAT), a marker of cholinergic neurons. Given the relevance of these habenular neurons in nicotine reinforcement and aversion [15, 16], we then proceeded to examine the effects of our new developed GPR3 agonist, RTI-19318-32 [17], on nicotine intake in wildtype mice. Since a therapeutic approach should be validated in consideration of the different levels of nicotine product use in human smokers, we examined mice across a range of low to high self-administered nicotine doses, specifically the lower 0.03 mg/kg/infusion, moderate 0.1 mg/kg/infusion and higher 0.25 mg/kg/infusion doses. For the low nicotine dose, the GPR3 agonists exerted a dose-dependent effect (Repeated measures (RM) one-way ANOVA, F_(2, 12)_ = 31.64, *p* < 0.0001). The post-hoc analysis revealed a reduction in nicotine intake in mice pretreated with RTI-19318-32 for both the 1 mg/kg (p = 0.003) and 10 mg/kg (p < 0.0001) doses of RTI-19318-32, as compared to the vehicle treated group (Fig. 1b). A difference in the number of lever presses was also found with RTI-19318-32 treatment (RM two-way ANOVA, *Lever* F_(2, 18)_ = 2.239, p = 0.1354*; Dose* F_(1, 18)_ = 54.85, *p* < 0.0001; *Interaction* F_(2, 18)_ = 2.292, p = 0.1298), with the post-hoc revealing a specific reduction in active lever presses at the 10 mg/kg RTI-19318-32 dose compared to vehicle (p = 0.0119) (Fig. 1c). For the moderate nicotine dose, the number of nicotine rewards was decreased with RTI-19318-32 treatment (RM one-way ANOVA, F_(2, 12)_ = 31.63, *p* < 0.0001), and the post-hoc revealed a statistically significant decrease in the number of rewards earned for both 1 mg/kg (p < 0.0001) and 10 mg/kg (p < 0.0001) RTI-19318-32 doses, as compared to vehicle (Fig. 1d). A selective reduction in active lever presses was also found at the moderate nicotine dose (RM two-way ANOVA, *Lever* F_(2, 18)_ = 15.44, p = 0.0001; *Dose* F_(1, 18)_ = 119.6, p < 0.0001; *Interaction* F_(2, 18)_ = 15.45, p = 0.0001), with the post-hoc indicating reduced active lever pressing behavior at both the 1 mg/kg (p < 0.0001) and 10 mg/kg (p < 0.0001) RTI-19318-32 doses (Fig. 1e). Finally, a high nicotine dose was examined to determine if these effects would be maintained with subjects that have the opportunity to self-administer larger amounts of nicotine. Treatment with RTI-19318-32 led to a reduction in nicotine intake (RM one-way ANOVA, F_(2, 12)_ = 18.83, p = 0.0002), which was found for both the 1 mg/kg (p = 0.0002) and 10 mg/kg (p = 0.0006) doses (Fig. 1f). Active, but not inactive, lever pressing was also decreased (RM two-way ANOVA, *Lever* F_(2, 18)_ = 17.35, *p* < 0.0001; *Dose* F_(1, 18)_ = 62.23, *p* < 0.0001; *Interaction* F_(2, 18)_ = 10.01, p = 0.0012) for the 1 mg/kg (p < 0.0001) and 10 mg/kg (p < 0.0001) RTI-19318-32 doses (Fig. 1g). Together, these data validate the GPR3 agonist approach as a viable therapeutic agent to reduce nicotine use in individuals consuming a range of daily nicotine doses.

### Limited off-target effects of the GPR3 agonist

**Fig. 2:**
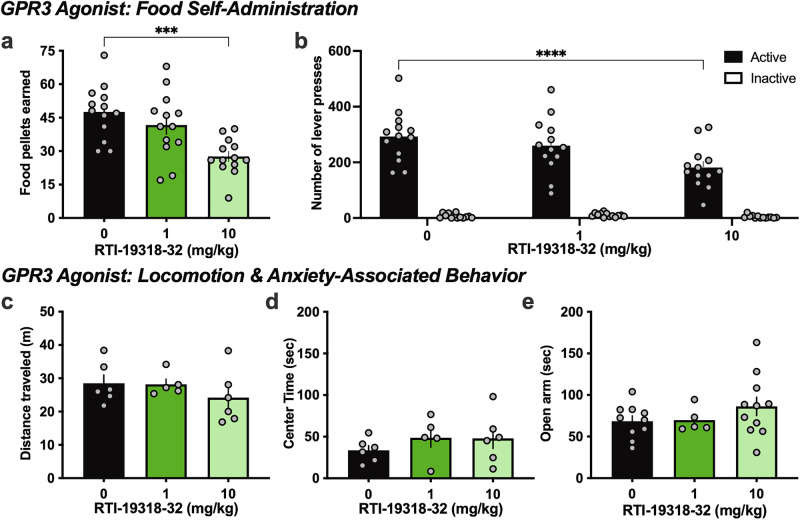
Minimal effects of the GPR3 agonist on food self-administration and anxiety-associated behaviors. **a** Treatment with RTI-19318-32 prior to a food self-administration session resulted in a reduction in the number of food pellets earned at the 10 mg/kg, but not 1 mg/kg, dose (n = 13 male mice). **b** The 10 mg/kg, but not 1 mg/kg, RTI-19318-32 dose selectively decreased lever pressing behavior on the active lever, and no differences were found among treatment groups on the inactive lever. In the open field test, administration of RTI-19318-32 had no effect on locomotion (**c**) or time spent in the center (**d**) (n = 5-6/treatment group, male mice). **e** In the elevated plus maze, RTI-19318-32 did not affect the time spent in the open arms, suggesting a lack of effect on anxiety-associated behavior (n = 5–11/treatment group, male mice). Data represented as mean ± SEM; all individual data points shown on bar figures. ***p < 0.001, ****p < 0.0001.

To determine whether the effects of the GPR3 agonist, RTI-19318-32, were specific to nicotine, we examined its effects on the animals’ ability to press a lever for food reward. After the mice achieved a stable level of food responding, RTI-19318-32 was administered prior to a session, and we found a reduction in the number of food pellets earned (RM one-way ANOVA F_(2, 24)_ = 12.70, p = 0.0002), but only at the higher 10 mg/kg dose of RTI-19318-32 (p = 0.0001) (Fig. 2a). In examining lever pressing behavior, a similar effect was found with the active lever responses (RM two-way ANOVA, *Lever* F_(2, 36)_ = 5.525, p = 0.0081, *Dose* F_(1, 36)_ = 273.4, p < 0.0001; *Interaction* F_(2, 36)_ = 4.894, p = 0.0132), in which the post-hoc analysis revealed decreased active lever pressing only at the high 10 mg/kg RTI-19318-32 dose (p < 0.0001) (Fig. 2b). Next, to provide additional insight into the effects of GPR3 on general feeding behavior, we examined whether acute administration of RTI-19318-32 would alter free feeding behavior or body weight in the home cage environment with 2 h daily feeding sessions. No differences were found in either the total amount of food consumed or body weight for both male and female mice following administration of 10 mg/kg RTI-19318-32 (Fig S1a, b). To further confirm that the effects of the GPR3 agonist were specifically related to reinforcement behavior, rather than general locomotor effects, we next examined subjects in the open field and elevated plus maze following treatment with RTI-19318-32. For the open field, no differences were found with agonist treatment in the distance traveled (One-way ANOVA, F _(2, 14)_ = 0.8332, p = 0.4551) (Fig. 2c) or in the time spent in the center of the open field (One-way ANOVA, F _(2, 14)_ = 0.7302, p = 0.4993) (Fig. 2d). RTI-19318-32 also did not alter the time spent on the open arms of the elevated plus maze (One-way ANOVA, F _(2, 23)_ = 1.230, p = 0.3109) (Fig. 2e). Together, these findings indicate that the GPR3 agonist does not alter general locomotor or anxiety-associated behaviors, although it may affect feeding-related behavior at the higher, but not lower, dose.

### GPR3 knockout results in increased nicotine intake in males, but not females

**Fig. 3:**
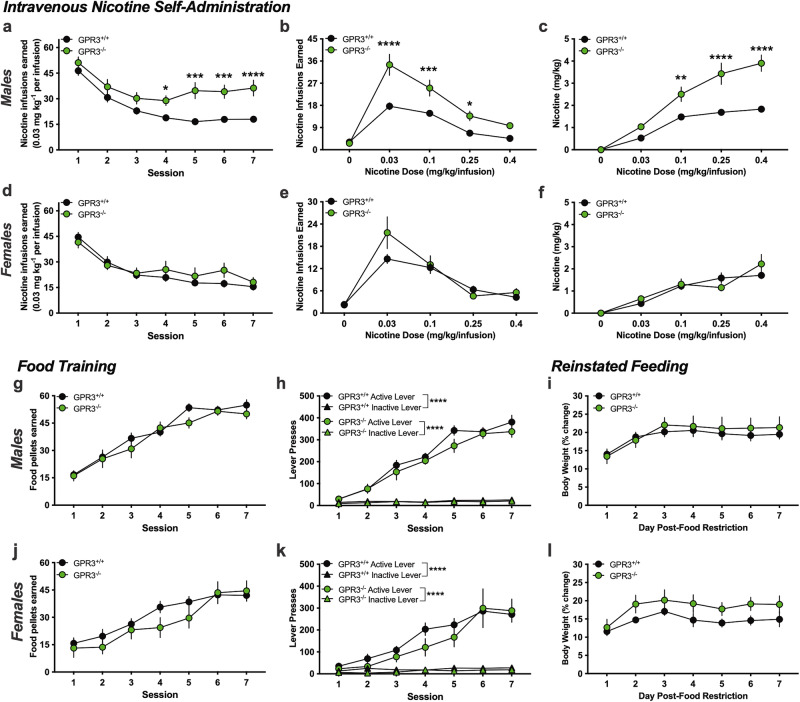
Male GPR3 knockout mice self-administer more nicotine than wildtype mice. **a** Acquisition of nicotine self-administration in male GPR3 knockout (GPR3^-/-^) and wildtype littermates (GPR3^+/+^) at the 0.03 mg/kg/infusion nicotine dose (n = 8–12/group). Across the baseline stabilization days 4–7, the male GPR3^-/-^ mice exhibited higher responding for nicotine infusions. **b** Male mice were examined across the dose response function, and the mean of the final 3 days on each dose was calculated. GPR3^-/-^ mice earned a greater number of nicotine infusions across low to moderate doses of nicotine (n = 7–8/group). No differences were found in the control condition with saline responding. **c** Data were transformed to determine the total amount of nicotine consumed at each dose during the 1 h sessions. Across the moderate to high doses, the male GPR3^-/-^ mice demonstrated high levels of nicotine intake beyond the maximum amount self-administered by a GPR3^+/+^ mouse. **d** In contrast, the female GPR3^-/-^ mice did not differ from their GPR3^+/+^ counterparts, in which both groups exhibited a similar level of self-administration during acquisition and baseline responding at the 0.03 mg/kg/infusion nicotine dose (n = 7-10/group). **e** Female GPR3^-/-^ and GPR3^+/+^ mice exhibited similar responding across the nicotine dose response function and also did not differ in their level of lever pressing for the saline control (n = 5-6/group). **f** The amount of nicotine consumed at each dose was calculated, and no differences were found between the female GPR3^-/-^ and GPR3^+/+^ groups. Male GPR3^-/-^ and GPR3^+/+^ mice acquired operant food responding similarly and did not differ in their number of food pellets earned within each session (**g**) or in their preference for the active versus inactive lever (**h**) (n = 10-21/group). (**i**) Male mice were examined for their percent change in free feeding body weight after a period of food restriction. No differences were found between the GPR3^-/-^ and GPR3^+/+^ groups in each session (n = 6-13/group). Female GPR3^-/-^ and GPR3^+/+^ mice acquired operant food responding similarly and did not differ in their number of food pellets earned within each session (**j**) or in their preference for the active versus inactive lever (**k**) (n = 8-11/group). **l** Female mice were examined for their percent change in free feeding body weight after a period of food restriction, and no differences between GPR3^-/-^ and GPR3^+/+^ groups were found (n = 5/group). Data represented as mean ± SEM. *p < 0.05, **p < 0.01, ***p < 0.001, ****p < 0.0001.

To further investigate the role of GPR3 in mediating nicotine reinforcement, male and female mice with null mutation in the GPR3 gene (GPR3^-/-^) were examined in comparison to wildtype littermates (GPR3^+/+^) with intravenous nicotine self-administration. Male mice acquired nicotine self-administration at the lower 0.03 mg/kg/infusion dose (RM two-way ANOVA, *Session* F _(6, 108)_ = 35.97, p < 0.0001; *Genotype* F _(1, 18)_ = 15.31, p = 0.001; *Interaction* F _(6, 108)_ = 4.021, p = 0.0011) (Fig. 3a). But, interestingly, male GPR3^-/-^ mice self-administered a significantly larger number of nicotine infusions than GPR3^+/+^ mice across sessions 5 (p < 0.0001), 6 (p = 0.0006) and 7 (p < 0.0001). Male mice were then examined across the full dose response function; the number of rewards differed based on dose and genotype (RM two-way ANOVA, *Dose* F _(4, 52)_ = 81.36, p < 0.0001; *Genotype* F _(1, 13)_ = 20.13, p = 0.0006; *Interaction* F _(4, 52)_ = 9.184, p < 0.0001) (Fig. 3b). Post-hoc analysis revealed that male GPR3^-/-^ mice earned a higher number of nicotine rewards at the 0.03 mg/kg/infusion (p < 0.0001), 0.1 mg/kg/infusion (p = 0.0008), and 0.25 mg/kg/infusion (p = 0.0408) doses. When these data were converted to determine the total amount of nicotine consumed at each dose in their 1 hr sessions, significant differences were found across the moderate to higher nicotine doses (RM two-way ANOVA, *Dose* F _(4, 52)_ = 82.13, p < 0.0001; *Genotype* F _(1, 13)_ = 32.55, p < 0.0001; *Interaction* F _(4, 52)_ = 10.16, p < 0.0001) (Fig. 3c), with the male GPR3^-/-^ mice self-administering higher mg/kg levels than the male GPR3^+/+^ mice at 0.1 mg/kg/infusion (p = 0.0063), 0.25 mg/kg/infusion (p < 0.0001) and 0.4 mg/kg/infusion (p < 0.0001). Surprisingly, the female GPR3^-/-^ did not differ from female GPR3^+/+^ mice when examined across all measures, including the acquisition of nicotine (RM two-way ANOVA, *Session* F _(6, 90)_ = 28.76, p < 0.0001, *Genotype* F _(1, 15)_ = 0.5820, p = 0.4574, *Interaction* F _(6, 90)_ = 1.357, p = 0.2405) (Fig. 3d), number of infusions across doses (RM two-way ANOVA, *Dose* F _(4, 36)_ = 27.95, p < 0.0001; *Genotype* F _(1, 9)_ = 0.981, p = 0.3478; *Interaction* F _(4, 36)_ = 1.822, p = 0.1460) (Fig. 3e), or total amount of nicotine consumed (RM two-way ANOVA, *Dose* F _(4, 36)_ = 25.80, p < 0.0001*; Genotype* F _(1, 9)_ = 0.5235, p = 0.4877; *Interaction* F _(4, 36)_ = 1.307, p = 0.2860) (Fig. 3f).

### No effect of GPR3 knockout on food reinforcement

To further validate the knockout findings and in consideration of the effects of the higher dose of the GPR3 agonist on food responding, we next examined whether GPR3^-/-^ and GPR3^+/+^ mice differ in their operant food self-administration behavior. No differences were found in the acquisition of food self-administration between genotypes in males (RM two-way ANOVA, *Session* F _(6, 174)_ = 72.35, p < 0.0001*; Genotype* F _(1, 29)_ = 0.8379, p = 0.3676*; Interaction* F _(6, 174)_ = 1.293, p = 0.2626) (Fig. 3g) or females (RM two-way ANOVA, *Session* F _(6, 102)_ = 30.48, p < 0.0001*; Genotype* F _(1, 17)_ = 0.8877, p = 0.3593*; Interaction* F _(6, 102)_ = 1.442, p = 0.2058) (Fig. 3j). For the males, a significant main effect of session and lever was found (RM two-way ANOVA, *Session* F _(6, 348)_ = 96.93, p < 0.0001; *Lever* F _(3, 58)_ = 113.1, p < 0.0001*, Interaction* F _(18, 348)_ = 35.29, p < 0.0001) (Fig. 3h), in which the post-hoc revealed that GPR3^+/+^ and GPR3^-/-^ mice discriminated between the active and inactive lever beginning on the second and third session, respectively, and continuing until the final 7th session (Session 3, 4, 5, 6 and 7, p < 0.0001). Although male GPR3^-/-^ mice had a lower number of active lever presses in session 5 (p = 0.0188), the groups did not differ in any of the other sessions or in the total number of food pellets earned across sessions. Female GPR3^-/-^ and GPR3^+/+^ groups acquired food training on the active and inactive levers in a similar manner (RM two-way ANOVA, *Session* F _(6, 204)_ = 39.39, p < 0.0001; *Lever* F _(3, 34)_ = 21.85, p < 0.0001*, Interaction* F _(18, 204)_ = 11.84, p < 0.0001) (Fig. 3k). Post-hoc analysis revealed that the female GPR3^+/+^ mice significantly discriminated between the active and inactive lever starting on day 3 (p = 0.0272), while GPR3^-/-^ mice exhibited lever discrimination on day 4 (p = 0.0338). This discrimination continued across all subsequent sessions for both genotypes (p < 0.001). In comparing active lever presses based on genotype, GPR3^-/-^ mice did not differ from GPR3^+/+^ mice. These data demonstrate that both the GPR3^-/-^ and GPR3^+/+^ male and female mice are able to learn to press a lever for food reward, discriminate between active and inactive levers, and achieve a similar number of food rewards when fully trained in the operant task.

Since GPR3 was previously shown to play a role in energy expenditure in mice [18], we next assessed for differences in body weight gain following food restriction when provided unlimited standard chow. No differences between GPR3^-/-^ and GPR3^+/+^ mice were found in their percentage of body weight gained with free feeding access for both the males (RM two-way ANOVA, *Day* F _(6, 102)_ = 41.27, p < 0.0001; *Genotype* F _(1, 17)_ = 0.1480, p = 0.7052; *Interaction* F _(6, 102)_ = 2.176, p = 0.0513) (Fig. 3i) and females (RM two-way ANOVA, *Day* F _(6, 48)_ = 10.23, p < 0.0001; *Genotype* F _(1, 8)_ = 2.396, p = 0.1603; *Interaction* F _(6, 48)_ = 0.9446, p = 0.4724) (Fig. 3l).

### Validation of the specificity of the GPR3 agonist

**Fig. 4:**
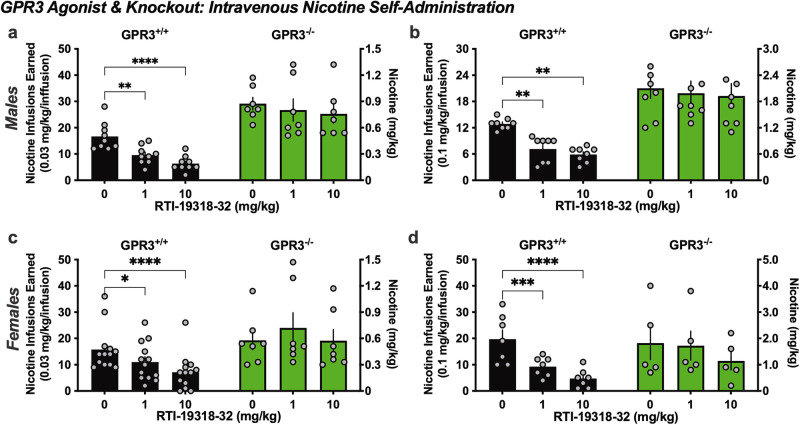
Specificity of the GPR3 agonist as validated in GPR3^-/-^ and GPR3^+/+^ mice. GPR3^-/-^ and GPR3^+/+^ mice were examined for the effects of GPR3 agonist RTI-19318-32 on intravenous nicotine self-administration. Graphs show both the number of nicotine infusions earned (left y-axis) and total mg/kg nicotine consumed (right y-axis). **a** Similar to that previously shown, male GPR3^-/-^ mice exhibited a higher level of responding for the 0.03 mg/kg/infusion dose of nicotine than GPR3^+/+^ mice. Both the 1 and 10 mg/kg dose of RTI-19318-32 were effective in reducing nicotine intake in the GPR3^+/+^ mice, but no differences were found in the GPR3^-/-^ mice (n = 7–9/group). **b** At the higher 0.1 mg/kg/infusion dose, both the 1 and 10 mg/kg dose of RTI-19318-32 were effective in reducing nicotine intake in the male GPR3^+/+^ mice, but RTI-19318-32 was without effect in the GPR3^-/-^ mice (n = 8/group). **c** At the lower 0.03 mg/kg/infusion nicotine dose in females, both the 1 and 10 mg/kg dose of RTI-19318-32 were effective in reducing nicotine intake in the female GPR3^+/+^ mice, but not in the female GPR3^-/-^ mice (n = 7-13/group). **d** At the higher 0.1 mg/kg/infusion dose, RTI-19318-32 at both 1 and 10 mg/kg were effective in reducing nicotine intake the female GPR3^+/+^, but not female GPR3^-/-^, mice (n = 5-7/group). Data represented as mean ± SEM; all individual data points shown on bar figures. *p < 0.05, **p < 0.01, ***p < 0.001, ****p < 0.0001.

As a further step to ensure rigor of our experimental findings, we next assessed the effects of the GPR3 agonist, RTI-19318-32, on nicotine self-administration in the GPR3^-/-^ and GPR3^+/+^ male and female mice for the low and moderate self-administered nicotine doses. For the males with the lower 0.03 mg/kg/infusion nicotine dose, a significant difference was found based on dose and genotype (RM two-way ANOVA, *Dose* F _(2, 28)_ = 11.82, p = 0.0002; *Genotype* F _(1, 14)_ = 30.43, p < 0.0001; *Interaction* F _(2, 28)_ = 2.525, p = 0.0982) (Fig. 4a). The post-hoc test demonstrated that we replicated and confirmed our prior findings, in which RTI-19318-32 reduced nicotine self-administration in the GPR3^+/+^ mice at both the 1 mg/kg (p = 0.0033) and 10 mg/kg (p < 0.0001) doses, while the GPR3^-/-^ mice were resistant to the effects of RTI-19318-32 at both doses. For the moderate 0.1 mg/kg/infusion dose of nicotine, the male GPR3^+/+^ and GPR3^-/-^ mice again differed by dose and genotype (RM two-way ANOVA, *Dose* F _(2, 28)_ = 6.406, p = 0.0051; *Genotype* F _(1, 14)_ = 23.91 p = 0.0002; *Interaction* F _(2, 28)_ = 2.482, p = 0.1018) (Fig. 4b). The post-hoc analysis replicated our prior findings with the male GPR3^+/+^ mice earning fewer nicotine infusions with RTI-19318-32 for both the lower 1 mg/kg (p = 0.0113) and higher 10 mg/kg (p = 0.0019) doses. However, no differences with RTI-19318-32 administration were found in the GPR3^-/-^ mice. These findings indicate that the observed effects of RTI-19318-32 in reducing nicotine intake are through GPR3 targeted mechanisms.

Female mice were then assessed in these studies to determine if the GPR3 agonist acts in a sex-specific manner given that the initial studies were conducted in male mice. For the females at the lower 0.03 mg/kg/infusion nicotine dose, significant main and interaction effects were found (RM two-way ANOVA, *Dose* F_(2, 36)_ = 7.310, p = 0.0022; *Genotype* F _(1, 18)_ = 5.391, p = 0.0322; *Interaction* F _(2, 36)_ = 7.803 p = 0.0015) (Fig. 4c). The post-hoc analysis revealed decreased nicotine intake with administration of RTI-19318-32 at 1 mg/kg (p = 0.0125) and 10 mg/kg (p < 0.0001) in the female GPR3^+/+^ mice. However, the GPR3 agonist was ineffective in altering nicotine intake in the female GPR3^-/-^ mice. For females self-administering higher levels at the 0.1 mg/kg/infusion nicotine dose, a significant difference was also found (RM two-way ANOVA, *Dose* F _(2, 20)_ = 18.59, p < 0.0001; *Genotype* F_(1, 10)_ = 0.8581, p = 0.3761; *Interaction* F _(2, 20)_ = 4.109, p = 0.032) (Fig. 4d). The post-hoc analysis of the female GPR3^+/+^ mice revealed a decrease in nicotine intake with 1 mg/kg (p = 0.0006) and 10 mg/kg (p < 0.0001) doses of RTI-19318-32. Moreover, the effects of the 1 and 10 mg/kg RTI-19318-32 doses did not differ from one another with any of the above analyses, indicating that they were equally effective in reducing nicotine intake. Taken together, these findings indicate that RTI-19318-32 is effective in both male and female mice to attenuate nicotine intake when subjects are consuming lower or higher levels of daily nicotine through actions on the GPR3 receptor.

### GPR3 localization in medial habenula neurons

**Fig. 5:**
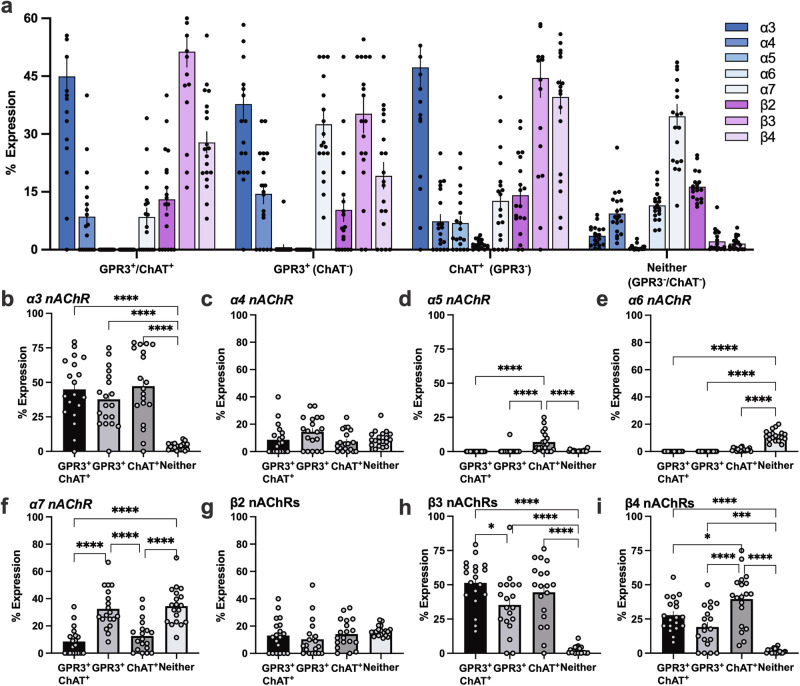
GPR3 is highly expressed in medial habenular neurons that co-express specific nicotinic acetylcholine receptors (nAChRs). Single cell sequencing data from medial habenular neurons of mice (n = 7 males, 12 females). **a** Neurons were quantified based on the expression of GPR3/ChAT co-expression, GPR3 (no ChAT), ChAT (no GPR3), and neither GPR3 nor ChAT. The resulting mean number of cells (±SEM) quantified in the medial habenula was as follows: ChAT/GPR3 (44 ± 9 cells), GPR3 (12 ± 2), ChAT (286 ± 44 cells), and neither (no ChAT nor GPR3) (1415 ± 376 cells). Graph shows the percentage of cells expressing each nAChR subunit (α3; α4; α5; α6; α7; β2; β3; β4) in the medial habenula based on the above main 4 group categorizations. **b**–**i** Percentage of categorized cells expressing individual nAChR subunits. **b** The α3 nAChR subunit was found to be highly expressed in GPR3, ChAT and GPR3/ChAT co-expressing cells, as compared to cells without either of these markers. **c** The α4 nAChR subunit exhibited a similar level of expression across cell categories. **d** The α5 nAChR subunit was found to be co-expressed in ChAT cells that did not express GPR3. **e** The α6 nAChR subunit was found in cells that did not express GPR3, and low levels in ChAT only cells. **f** The α7 nAChR subunit was localized in all cell populations, with highest co-expression in the GPR3 cells that did not express ChAT and the cells without either GPR3 or ChAT. **g** Similar co-expression of the β2 nAChR subunit was found among all cell categories. **h** High co-expression of the β3 nAChR subunit was found in cells expressing GPR3 and/or ChAT. **i** The β4 nAChR subunit exhibited the highest percentage of co-expression in ChAT cells, followed by those that co-express GPR3 either with or without ChAT. Data represented as mean ± SEM; all individual data points shown on bar figures. *p < 0.05, ***p < 0.001, ****p < 0.0001.

To provide insight into the medial habenula cells that are putatively being affected by GPR3 agonist treatment, we then assessed expression of the different nAChR subunits across cell populations. Analysis of open source single cell data from the mouse medial habenula, in which cells were transcriptionally defined based on the Allen Mouse Brain Common Coordinate Framework [21], differential expression of nAChR subunits was revealed based on the following categorized subpopulations: (1) co-expressing GPR3/ChAT cells (GPR3^+^/ChAT^+^), (2) GPR3 expressing cells, no ChAT (GPR3^+^), (3) ChAT expressing cells, no GPR3 (ChAT^+^), and (4) expressing neither GPR3 nor ChAT (neither, GPR3^-^/ChAT^-^) (Fig. 5a). The total number of cells for each of the above categorized subpopulations was used as the denominator to determine percent co-expression. For the α3 nAChR subunit, significant differences were found in expression levels across cell types (One-way ANOVA, F _(3, 72)_ = 19.71, p < 0.0001) (Fig. 5b). The post-hoc analysis revealed that α3 was highly expressed in the GPR3^+^/ChAT^+^ co-expression (p < 0.0001), GPR3^+^ (p < 0.0001), and ChAT^+^ (p < 0.0001) cells, as compared to cells lacking expression of GPR3 and ChAT. For the α4 nAChR subunit, no significant differences were found among groups (One-way ANOVA, F _(3, 72)_ = 2.196, p = 0.0959) (Fig. 5c). For the α5 nAChR subunit, expression was present in ChAT^+^ cells, but unexpectedly, not at significant levels for all other categorized groups (One-way ANOVA, F _(3, 72)_ = 12.05, p < 0.0001; post-hoc, ChAT^+^ vs GPR3^+^/ChAT^+^ p < 0.0001; ChAT^+^ vs GPR3^+^ p < 0.0001; ChAT^+^ vs GPR3^-^/ChAT^-^ p < 0.0001) (Fig. 5d). The α6 nAChR subunit displayed predominant expression in cells lacking ChAT and GPR3 cells, with little to no expression in the other cell categories (F _(3, 72)_ = 134.4, p < 0.0001; post-hoc, GPR3^+^/ChAT^+^ vs GPR3^-^/ChAT^-^ p < 0.0001; GPR3^+^ vs GPR3^-^/ChAT^-^ p < 0.0001; ChAT^+^ vs GPR3^-^/ChAT^-^ p < 0.0001) (Fig. 5e). For the α7 nAChR subunit, high levels of expression were found in cells not expressing ChAT, including the GPR3 only populations (F _(3, 72)_ = 21.55, p < 0.0001; post-hoc, GPR3^+^/ChAT^+^ vs GPR3^+^ p < 0.0001; GPR3^+^/ChAT^+^ vs GPR3^-^/ChAT^-^ p < 0.0001; GPR3^+^ vs ChAT^+^ p < 0.0001; ChAT^+^ vs GPR3^-^/ChAT^-^ p < 0.0001) (Fig. 5f). For the β2 nAChR subunit, no differences were found across cell populations examined (One-way ANOVA, F _(3, 72)_ = 1.050, p = 0.3760) (Fig. 5g). The β3 nAChR subunit was preferentially localized in cells expressing GPR3 and/or ChAT (One-way ANOVA, F _(3, 72)_ = 28.54, p < 0.0001; post-hoc, GPR3^+^/ChAT^+^ vs Gpr3^+^ p = 0.0336; GPR3^+^/ChAT^+^ vs GPR3^-^/ChAT^-^ p < 0.0001; GPR3^+^ vs GPR3^-^/ChAT^-^ p < 0.0001; ChAT^+^ vs GPR3^-^/ChAT^-^ p < 0.0001) (Fig. 5h). Finally, the β4 nAChR subunit was found to be preferentially expressed in GPR3 and/or ChAT cells, with the highest level of expression in the only ChAT-positive cells (One-way ANOVA, F _(3, 72)_ = 26.19, p < 0.0001; post-hoc, ChAT^+^/GPR3^+^ vs ChAT^+^ p = 0.0452; ChAT^+^/GPR3^+^ vs GPR3^-^/ChAT^-^ p < 0.0001; GPR3^+^ vs ChAT^+^ p < 0.0001; GPR3^+^ vs GPR3^-^/ChAT^-^ p = 0.0009; ChAT^+^ vs GPR3^-^/ChAT^-^ p < 0.0001) (Fig. 5i). Data were compiled for both males and females given that no significant sex differences were identified in examining GPR3-expressing cells with the single cell transcriptomic data, and further, no differences were found between adult male and female GPR3 mRNA expression in the medial habenula with RT-qPCR (Fig S1c and S2). In consideration of the sex differences found in the GPR3^-/-^ mice for nicotine acquisition, we further assessed whether this could be explained by sex differences in the co-expression of GPR3 with *Esr1* (estrogen receptor α), *Esr2* (estrogen receptor β), and Gper1 (G protein-coupled estrogen receptor), but no significant differences were found in the medial habenula (Fig S1d). Taken together, these findings reveal that GPR3-expressing cells co-localize with numerous nAChR subunits that can form a variety of functional nAChR subtypes. Based on the patterns evidenced, it indicates that GPR3 modulation most likely affects cholinergic neurons containing the α3β4* nAChRs to impact nicotine intake, which is consistent with our prior findings demonstrating the involvement of medial habenular α3β4*cells as critical regulators of nicotine reinforcement [16]. Given the co-expression patterns, it is also likely that the α3β4* nAChR cell population has functional α3β3β4 nAChRs [22, 23], although this specific receptor subtype in the habenula has not yet been directly shown to alter intravenous nicotine self-administration.

## Discussion

Here, we provide extensive evidence validating GPR3 receptor targeting as a novel therapeutic approach for nicotine cessation. Our newly developed GPR3 agonist, RTI-19318-32, was found to decrease nicotine self-administration in both male and female wildtype mice. This effect persisted across the low, moderate, and high doses of nicotine. The low dose of the agonist had no effect on food training or overall responses for food reward, suggesting specificity in mediating nicotine reinforcement. Control behavioral analysis further supported the selectivity of GPR3 agonist action, with no off-target effects of RTI-19318-32 on general locomotor behavior and anxiety-associated behavior. Moreover, the specificity of RTI-19318-32 for its actions on the GPR3 receptor was demonstrated by a lack of effect in altering nicotine intake in male and female GPR3 knockout mice. Given that prior studies of GPR3 either used male mice only [24–26] or did not report the sex of the subjects [27, 28], the effectiveness of the GPR3 agonist in females represents an important finding to support therapeutic use potential for the general population. Finally, GPR3 expression co-localized with multiple nAChR subunits in the medial habenula, and given our prior findings demonstrating the involvement of medial habenular α3β4* nAChRs in nicotine reinforcement [16], the current findings suggest that these co-expressing cells may underlie the behavioral effects evidenced. Taken together, these studies provide evidence that pharmacological targeting to activate the GPR3 receptor may be an effective therapeutic approach for both sexes and in subjects with varying levels of daily nicotine use.

### GPR3 knockout mouse model

GPR3 is expressed in the CNS, as well as in peripheral adipocytes [13, 24]. In the brain, GPR3 has been localized to the medial habenula, striatum, cortex, hippocampus, and thalamus [13]. Given this expression profile, initial investigations with the GPR3^-/-^ mice were focused on emotion-associated behaviors and cocaine intake. GPR3^-/-^ mice were previously shown to self-administer higher amounts of cocaine during part of the acquisition period, but no phenotypic differences were found once stable responding was achieved [29]. Another study demonstrated that GPR3^-/-^ mice exhibited higher levels of anxiety-associated behavior in the elevated plus maze and depression-like behavior in the forced swim and tail suspension tests [25]. These behavioral findings in male mice were then correlated with altered monoamine neurotransmitter levels, which found a reduction in serotonin in the hippocampus, hypothalamus and frontal cortex, noradrenaline in the hypothalamus and frontal cortex, and dopamine in the hippocampus [25]. Therefore, one may predict that increasing GPR3 signaling could induce an opposing effect than that observed with the knockout model. For instance, the GPR3 agonist would be predicted to decrease anxiety- and depression-like behaviors based on this prior study, which could make the agonist an even more attractive target for nicotine therapeutics, as nicotine use is often associated with anxiety and depression in humans [30]. In our studies, we did not find any differences in anxiety-associated behaviors following administration of the GPR3 agonist, although it should be noted that the mice were tested at baseline and not in an anxiety-induced state. Importantly, the studies with the GPR3 knockout mice permitted us to (1) replicate our prior findings in the male wildtype mice in which RTI-19318-32 was found to reduce nicotine intake, (2) determine that RTI-19318-32 induced similar effects in reducing nicotine intake in female wildtype mice, and (3) determine that RTI-19318-32 was ineffective in altering drug intake in both male and female mice lacking GPR3 gene expression. Based on the effectiveness of our GPR3 agonist in both male and female mice with nicotine and the differential sex-specific effects of GPR3 knockout on nicotine behaviors, these findings reveal limitations of using this knockout model. Indeed, in consideration of the constitutive elimination of the *Gpr3* gene in the GPR3^-/-^ mouse model, compensatory changes across development could have led to some of the noted phenotypic differences found in adulthood. This contention is supported by the finding that GPR3 can regulate early neurodevelopment processes with outgrowth of cerebellar granule neurons and polarization of hippocampal neurons [26–28]. However, these prior neurodevelopmental findings are based on in vitro studies, and thus, it still remains unknown what specific effect GPR3 may exert in vivo during discrete developmental stages and how biological sex may intersect with these processes. Indeed, we hypothesized that co-expression of GPR3 and estrogen receptors within the same cells may have been a central factor in mitigating this interaction, but in adulthood, we could not find any differences in the expression of estrogen receptor α, estrogen receptor β, or the G protein-coupled estrogen receptor in the habenula between males and females. Thus, further studies are needed to more systematically examine this putative interaction in cells co-expressing GPR3 and the estrogen receptors across multiple brain regions and peripheral cell types, in addition to specifically ablating the expression of the GPR3 receptor in the medial habenula of the adult to demonstrate the specificity of potential site-specific involvement in nicotine-related behaviors.

### Implications of peripheral GPR3 expression

Outside of the CNS, GPR3 has been shown to be expressed in adipocytes and to play a role in thermogenesis and energy expenditure [18, 24, 31]. GPR3 activity increases cAMP levels in adipocytes to activate thermogenic mechanisms and increasing energy expenditure [24]. Rather than acting through canonical adrenergic mechanisms, GPR3 appears to be constitutively active and mainly controlled through transcriptional regulation [24]. This transcriptional regulation of GPR3 can be driven by lipolytic signals, which can become increased through cold exposure and high fat diet [24]. These prior findings support the notion that GPR3 may be a possible target for treating metabolic disease, especially considering that in humans, higher levels of GPR3 expression in brown adipose tissue was correlated with a lower body mass index [24]. Interestingly, the discontinuation of nicotine products has been associated with increased weight gain and adiposity, an effect that has also been recapitulated in animal models [32, 33]. Weight gain is also cited as one of the top reasons people relapse and continue using nicotine products [34]. However, we did not find any differences between the GPR3 wildtype and knockout mice in their body weight gain following food restriction under free chow access conditions. During nicotine self-administration, mice were maintained at the same target weight across all sessions for dosing with the GPR3 agonist, so the effects on nicotine intake were not secondary to altered body weight. Moreover, acute administration of RTI-19318-32 did not alter body weight or food consumption with 2 h daily free-feeding sessions. Thus, the GPR3 agonist-induced differences in nicotine self-administration cannot be attributed to feeding behavior or body weight based on the current evidence, but rather, are indicative of differences in reinforcement-driven behavior.

GPR3 exhibits constitutive activity both in vitro and ex vivo in CNS-derived cells [24, 25]. It is difficult, however, to demonstrate that the receptor is independently constitutively active, activated by a ubiquitous ligand, or a combination of both under endogenous conditions in vivo with current tools available. Both N-terminus mediated intrinsic signaling as well as oleic acid ligand induced activity have been reported [24, 35]. Oleic acid is abundant in the CNS and is a major constituent of membrane phospholipids in the brain [36]. Sources of oleic acid include dietary intake as well as synthesis by astrocytes [37, 38]. Thus, it is possible that GPR3 confers intrinsic adenylyl cyclase signaling in addition to lipid-mediated activity at the neuronal membrane. Future studies on the influence of oleic acid and GPR3 activity in vivo are needed to confirm the physiological relevance of this finding.

### Targeting GPR3 for nicotine cessation

Our data reveals the potential of GPR3 as a novel nicotine cessation drug target. Individuals vary greatly in their levels of daily nicotine intake, so it is important that a cessation aid is effective across a range of levels. It is also important to validate its efficacy in both males and females. We found that the GPR3 agonist was highly effective at reducing nicotine intake at low, moderate, and high nicotine doses in both males and females. Given that both GPR3 agonist doses exerted a very similar level of effectiveness with around a ~60% reduction, despite a 10x greater amount administered at the higher dose examined, there appears to be a wide therapeutic window with potentially minimal off-target actions. Of further note, this level of reduced responding for nicotine is greater than that found with rodent studies of the first line therapeutics, bupropion and varenicline, at moderate doses with ~30-45% reductions in intake [39–41]. Higher doses of bupropion and varenicline can induce greater reductions of nicotine intake yet can also be associated with adverse side effects and only moderate long-term effectiveness in reducing smoking behavior [8, 9, 39]. This highlights the significant need for the further development of cessation aids with varying mechanisms of action, which may have utility either alone, or in combination with the other currently available medications. However, one noted limitation of RTI-19318-32 is that the backbone of the compound is an NADPH oxidase inhibitor [42], which can accumulate in the mitochondria to potentially exert deleterious effects under chronic dosing conditions. As such, we were unable to repeatedly inject the mice for chronic treatment studies, and thus, further studies are currently directed at medicinal chemistry campaigns to derive a GPR3 agonist that can be administered chronically in humans. In sum, the current investigations reveal the functional significance of and validate GPR3 as a viable target for smoking cessation therapeutic development, which has the potential to provide a foundation to assist individuals seeking to improve their overall heath by quitting the use of nicotine-containing products.

## Supplementary information

Supplementary Figures

## Data Availability

The data that support the findings of this study are openly available at OSF (https://osf.io/dashboard) 10.17605/OSF.IO/UZFHN.
