# Supplementary material for: Targeting GPR3 as a novel approach for nicotine cessation therapeutic development
Source: Neuropsychopharmacology. 2025 Aug 27;50(13):2051–62. doi: 10.1038/s41386-025-02202-3 (PMC12603151; doi:10.1038/s41386-025-02202-3)
Supplement: Supplementary file 1 — Supplementary Figures [file 41386_2025_2202_MOESM1_ESM.pdf]

SUPPLEMENTARY FIGURES

Targeting GPR3 as a Novel Approach for Nicotine Cessation Therapeutic Development

Allison S. Mogul[1], Kendyl N. Laumann[1], Malia Bautista[1], JP Fowler[1], Bruce E. Blough[2], Elaine A. Gay[2], and Christie D. Fowler[1*]

[1]Department of Neurobiology and Behavior, University of California Irvine, Irvine, CA, 92617,USA
[2]Center for Drug Discovery, RTI International, Research Triangle Park, NC 27709, USA

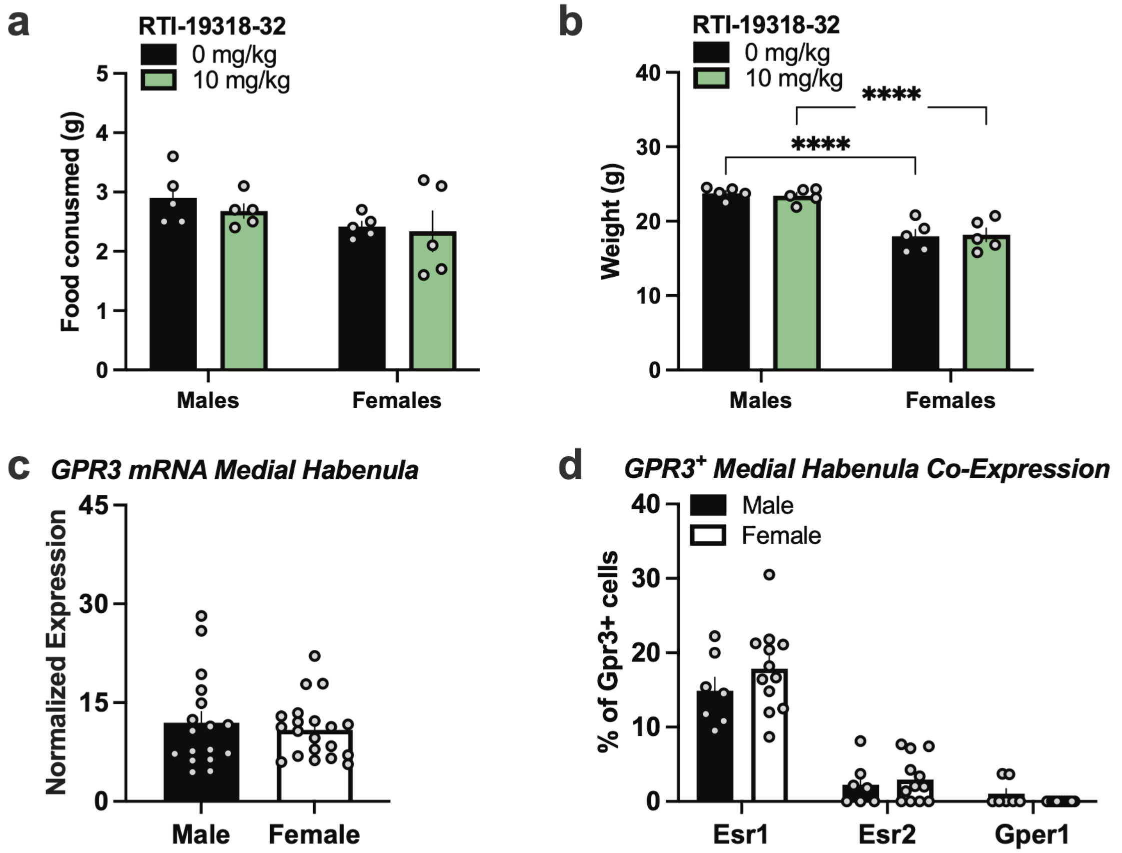

Supplementary Figure S1. Control analyses reveal lack of effect of the GPR3 agonist on feeding, and no sex differences in GPR3 gene expression and estrogen receptor co-expression. (**a-b**) Acute administration of RTI-19318-32 did not alter the amount of food consumed (**a**) or body weight (**b**) in males or females during daily 2 hr feeding sessions. Females were found to weigh less than males, but there was no interaction based on GPR3 agonist administration. (**a**: Two-way ANOVA, *Sex* $F_{(1, 8)}$=3.820 p=0.0864, *Dose* $F_{(1, 8)}$=0.4818 p=0.5073, *Interaction* $F_{(1, 8)}$=0.1049 p=0.7543; **b**: Two-way ANOVA, *Sex* $F_{(1, 8)}$=31.19 p=0.0005, *Dose* $F_{(1, 8)}$=0.7446 p=0.4133, *Interaction* $F_{(1, 8)}$ = 4.486 p=0.0670, post-hoc: 0 mg/kg males vs females ****p<0.0001, 10 mg/kg males vs females ****p<0.0001) (**c**) RT-qPCR reveals no differences in GPR3 mRNA expression between males and females for medial habenula tissue. *Gpr3* mRNA expression was normalized to β-actin mRNA levels. (t-test, $t_{(35)}$=0.5514 p=0.5849) (**d**) In examining the percentage of GPR3 cells that express the different estrogen receptors with the single cell open-source medial habenula dataset [21], no statistically significant differences were found between males and females for *Esr1*, *Esr2*, and *Gper1*. (Two-way ANOVA, *Sex* $F_{(1, 51)}$=0.7624 p=0.3867, *Receptor* $F_{(2, 51)}$=99.76 p<0.0001, *Interaction* $F_{(2, 51)}$=1.367, p=0.2642) Data represented as mean ± SEM; all individual data points shown on bar figures. ****p<0.0001.

a

Medial Habenula: Total Number of Cells Per Subject

Females	f10	f11	f12	f13	f14	f15	f16	f17	f18	f19	f21	f22
GPR3+/ChAT+	34	61	35	22	33	19	44	42	54	70	9	23
GPR3+ (ChAT-)	8	12	5	37	17	4	3	7	36	8	2	4
ChAT+ (GPR3-)	132	198	34	265	298	168	203	196	465	432	168	132
**Males**	**m11**	**m12**	**m13**	**m15**	**m16**	**m18**	**m22**					
GPR3+/ChAT+	34	14	201	44	29	62	15					
GPR3+ (ChAT-)	20	3	30	11	10	12	5					
ChAT+ (GPR3-)	272	60	1532	132	194	432	123					

b

nAChR Subunit	Sex	Cell Groups	% Cells Mean±SEM	N	Test	Factors	Statistic	P-Value	Posthoc	P-Value	Significance
**α3**	Females	GPR3+ ChAT+	43.26 ± 6.41	12	Two-way ANOVA	Interaction	F (2, 51) = 0.06287	P=0.9391			
		GPR3+	37.73 ± 5.72	12		Cell Type	F (2, 51) = 0.9062	P=0.4105			
		ChAT+	45.33 ± 6.53	12		Sex	F (1, 51) = 0.2640	P=0.6096			
	Males	GPR3+ ChAT+	47.84 ± 9.34	7							
		GPR3+	37.83 ± 8.23	7							
		ChAT+	50.60 ± 12.22	7							
**α4**	Females	GPR3+ ChAT+	8.43 ± 3.69	12	Two-way ANOVA	Interaction	F (2, 51) = 0.2419	P=0.7861			
		GPR3+	15.92 ± 3.31	12		Cell Type	F (2, 51) = 2.271	P=0.1136			
		ChAT+	8.66 ± 2.45	12		Sex	F (1, 51) = 0.6900	P=0.4100			
	Males	GPR3+ ChAT+	8.86 ± 2.85	7							
		GPR3+	12.01 ± 4.32	7							
		ChAT+	5.11 ± 2.34	7							
**α5**	Females	GPR3+ ChAT+	0 ± 0	12	Two-way ANOVA	Interaction	F (2, 51) = 4.198	P=0.0205	Šídák's multiple comparisons test		
		GPR3+	1.04 ± 1.04	12		Cell Type	F (2, 51) = 10.27	P=0.0002	Male - Female		
		ChAT+	9.76 ± 2.39	12		Sex	F (1, 51) = 6.123	P=0.0167	GPR3+ ChAT+	>0.9999	ns
	Males	GPR3+ ChAT+	0 ± 0	7					GPR3+	0.9417	ns
		GPR3+	0 ± 0	7					ChAT+	0.0013	**
		ChAT+	2.05 ± 1.00	7							
**α6**	Females	GPR3+ ChAT+	0 ± 0	12	Two-way ANOVA	Interaction	F (2, 51) = 0.2177	P=0.8051			
		GPR3+	0 ± 0	12		Cell Type	F (2, 51) = 22.28	P<0.0001			
		ChAT+	1.42 ± 0.35	12		Sex	F (1, 51) = 0.2177	P=0.6428			
	Males	GPR3+ ChAT+	0 ± 0	7							
		GPR3+	0 ± 0	7							
		ChAT+	1.16 ± 0.38	7							
**α7**	Females	GPR3+ ChAT+	10.89 ± 3.22	12	Two-way ANOVA	Interaction	F (2, 51) = 2.232	P=0.1177			
		GPR3+	28.61 ± 3.73	12		Cell Type	F (2, 51) = 23.64	P<0.0001			
		ChAT+	12.16 ± 3.12	12		Sex	F (1, 51) = 0.3128	P=0.5784			
	Males	GPR3+ ChAT+	4.44 ± 2.38	7							
		GPR3+	39.24 ± 6.02	7							
		ChAT+	13.53 ± 5.14	7							
**β2**	Females	GPR3+ ChAT+	13.86 ± 4.09	12	Two-way ANOVA	Interaction	F (2, 51) = 0.1117	P=0.8946			
		GPR3+	12.43 ± 4.61	12		Cell Type	F (2, 51) = 0.5743	P=0.5667			
		ChAT+	15.07 ± 3.21	12		Sex	F (1, 51) = 1.042	P=0.3121			
	Males	GPR3+ ChAT+	11.71 ± 3.25	7							
		GPR3+	6.77 ± 2.66	7							
		ChAT+	12.60 ± 3.43	7							
**β3**	Females	GPR3+ ChAT+	49.05 ± 4.69	12	Two-way ANOVA	Interaction	F (2, 51) = 3.629	P=0.0336	Šídák's multiple comparisons test		
		GPR3+	43.40 ± 5.23	12		Cell Type	F (2, 51) = 4.680	P=0.0136	Male - Female		
		ChAT+	40.52 ± 6.49	12		Sex	F (1, 51) = 0.08927	P=0.7663	GPR3+ ChAT+	0.8802	ns
	Males	GPR3+ ChAT+	55.31 ± 7.53	7					GPR3+	0.0660	ns
		GPR3+	21.36 ± 7.80	7					ChAT+	0.5754	ns
		ChAT+	51.46 ± 8.08	7							
**β4**	Females	GPR3+ ChAT+	27.90 ± 3.60	12	Two-way ANOVA	Interaction	F (2, 51) = 1.944	P=0.1536			
		GPR3+	25.11 ± 3.78	12		Cell Type	F (2, 51) = 9.801	P=0.0003			
		ChAT+	38.50 ± 5.81	12		Sex	F (1, 51) = 1.099	P=0.2994			
	Males	GPR3+ ChAT+	27.64 ± 4.72	7							
		GPR3+	9.00 ± 4.87	7							
		ChAT+	41.54 ± 7.14	7							

Supplementary Figure S2. Single cell data analyses for sex differences in the medial habenula. (**a**) Total number of medial habenula cells reported for each subject with the single cell analysis performed by Macosko and colleagues (accessible at: https://singlecell.broadinstitute.org) [21]. Subject ID for sex denoted as f: female, m: male. (**b**) Statistical table comparing males and females for the percent of GPR3$^+$/ChAT$^+$, GPR3$^+$ (ChAT$^-$), and ChAT$^+$ (GPR3$^-$) cells co-expressing each nAChR subunit indicated in the left column. Percent expression calculated based on the total number of cells in each of the individual cell type categories (GPR3$^+$/ChAT$^+$, GPR3$^+$ (ChAT$^-$), and ChAT$^+$ (GPR3$^-$)). For reference, the total cell number used as the denominator for percentage calculations is indicated above in panel (**a**) for each subject. The Sidak's multiple comparison post-hoc analysis was conducted following a statistically significant main effect of sex or interaction to permit comparison of male and female data as shown.
